# Absolute configuration of 3β-acet­oxy­olean-11,12-aziridin-28,13-β-olide

**DOI:** 10.1107/S1600536811014875

**Published:** 2011-04-29

**Authors:** Wen Nee Tan, Keng Chong Wong, Melati Khairuddean, Madhukar Hemamalini, Hoong-Kun Fun

**Affiliations:** a School of Chemical Sciences, Universiti Sains Malaysia, 11800 USM, Pulau Penang, Malaysia; b X-ray Crystallography Unit, School of Physics, Universiti Sains Malaysia, 11800 USM, Penang, Malaysia

## Abstract

The title compound, C_32_H_49_NO_4_, has been isolated from the dichloro­methane extract of the stem bark of *Garcinia atroviridis* Griff. ex T. Anders. Rings *A* and *B*, *B* and *C*, and *C* and *D* are *trans*-fused, whereas rings *D* and *E* are *cis*-fused. Rings *A*, *B*, *C* and *E* have slightly distorted chair conformations, while ring *D* is most heavily distorted towards a half-chair conformation owing to the strain induced by the lactonization. The ester group attached to ring *A* is in an equatorial position.

## Related literature

For details and applications of *Garcinia atroviridis* Griff. ex T. Anders, see: Permana *et al.* (2001 ▶); Amran *et al.* (2009 ▶). For bond-length data, see: Allen *et al.* (1987 ▶). For ring conformations, see: Cremer & Pople (1975 ▶). For the stability of the temperature controller used in the data collection, see: Cosier & Glazer (1986 ▶).
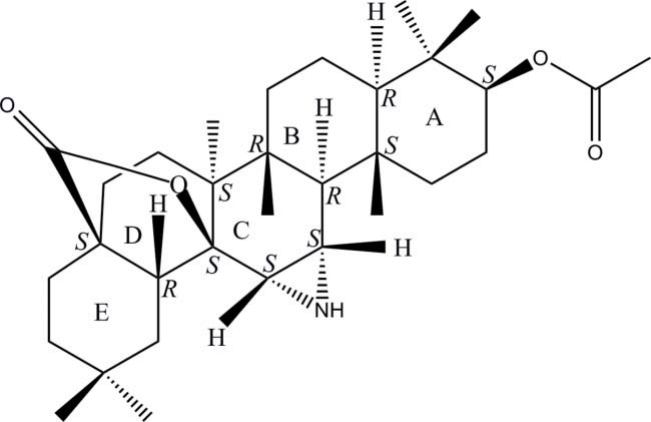


## Experimental

### 

#### Crystal data

C_32_H_49_NO_4_
*M* *_r_* = 511.72
Monoclinic, 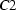

*a* = 13.0197 (2) Å
*b* = 6.7460 (1) Å
*c* = 32.0674 (5) Å
β = 100.6452 (4)°
*V* = 2768.04 (7) Å^3^
*Z* = 4
Cu *K*α radiation
μ = 0.62 mm^−1^
*T* = 100 K
0.73 × 0.15 × 0.14 mm

#### Data collection

Bruker SMART APEX DUO CCD area-detector diffractometer
Absorption correction: multi-scan (*SADABS*; Bruker, 2009 ▶) *T*_min_ = 0.659, *T*_max_ = 0.917
8407 measured reflections
3061 independent reflections
3050 reflections with *I* > 2σ(*I*)
*R*_int_ = 0.018

#### Refinement

*R*[*F* ^2^ > 2σ(*F* ^2^)] = 0.033
*wR*(*F* ^2^) = 0.087
*S* = 1.02
3061 reflections
342 parameters
1 restraint
H-atom parameters constrained
Δρ_max_ = 0.32 e Å^−3^
Δρ_min_ = −0.37 e Å^−3^
Absolute structure: Flack (1983 ▶), 721 Friedel pairs
Flack parameter: 0.1 (2)

### 

Data collection: *APEX2* (Bruker, 2009 ▶); cell refinement: *SAINT* (Bruker, 2009 ▶); data reduction: *SAINT*; program(s) used to solve structure: *SHELXTL* (Sheldrick, 2008 ▶); program(s) used to refine structure: *SHELXTL*; molecular graphics: *SHELXTL*; software used to prepare material for publication: *SHELXTL* and *PLATON* (Spek, 2009 ▶).

## Supplementary Material

Crystal structure: contains datablocks global, I. DOI: 10.1107/S1600536811014875/is2699sup1.cif

Structure factors: contains datablocks I. DOI: 10.1107/S1600536811014875/is2699Isup2.hkl

Additional supplementary materials:  crystallographic information; 3D view; checkCIF report

## Figures and Tables

**Fig. 1.**
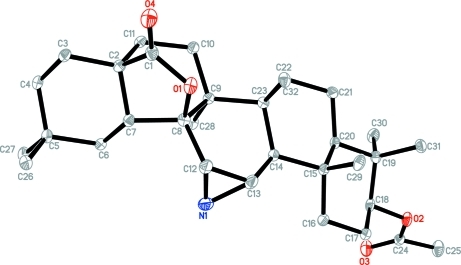
The asymmetric unit of the title compound. Displacement ellipsoids are drawn at the 30% probability level. H atoms omitted for clarity.

## supplementary crystallographic information

### Comment

*Garcinia atroviridis Griff. ex T. Anders.* (Clusiaceae) is a medium-sized fruit tree which may be found growing wild or cultivated throughout Peninsular Malaysia (Permana *et al.*, 2001). In folkloric medicine, it has been used as a postpartum medication agent as well as an agent to treat earache, throat irritation, cough, dandruff and some stomachache associated with pregnancy (Amran *et al.*, 2009). In our research on this plant, the stem bark extracts of *G. atroviridis* were examined. The title compound (I), 3β-acetoxyolean-11,12-aziridin-28,13-β-olide, has been isolated from the dichloromethane extract.

The title molecule presented in Fig. 1 contains five six-membered rings, namely, *A* (C15–C20), *B* (C14/C15/C20–C23), *C* (C9/C8/C12–C14/C23), *D* (C2/C7–C11) and *E* (C2–C7). The ester group attached to ring *A* is in an equatorial position. The bond distances (Allen *et al.*, 1987) and angles in (I) are as expected. Rings *A*/*B*, *B*/*C* and *C*/*D* are trans-fused, whereas rings *D*/*E* are cis-fused. Rings *A*, *B*, *C* and *E* have slightly distorted chair conformations, ring *D* being most heavily distorted towards a half-chair conformation due to the strain induced by the lactonization, as shown by the Cremer & Pople, (1975) parameters: [ring  *A*: Q = 0.572 (2) Å, θ = 175.5 (2)°  and φ = 325 (3)°; *B*: Q = 0.584 (2) Å, θ = 11.6 (2)° and f = 118.6 (10)°; *C*: Q = 0.574 (2) Å, θ = 46.9 (2)° and φ = 111.4 (3)°; *D*: Q = 0.629 (2) Å, θ = 166.06 (18)° and f = 236.5 (8); *E*: Q = 0.571 (2) Å, θ = 3.8 (2)° and f = 202 (4)°]. The absolute configurations of the natural product molecule were determined by the refinement of the Flack parameter to 0.1 (2). There are eleven chiral centres in the molecule. From the structure presented, these centers exhibit the following chiralities: C2 = S, C7 = R, C8 = S, C9  = S, C12 = S,  C13 = S, C14 = R, C15 = S, C18 = S, C20 = R and C23 = R. There are no classical hydrogen bond, weak interaction, *Cg*–*Cg* and C—H···π interactions in the crystal packing.

### Experimental

Air-dried stem bark of *G. atroviridis* was ground and sequentially extracted in a Soxhlet apparatus with hexane, dichloromethane and methanol. The dichloromethane extract after concentration was subjected to silica gel column chromatography using a hexane-chloroform-ethylacetate-methanol gradient to afford 58 fractions (D1–D58). Fraction D8 was further fractionated with hexane-ethyl acetate gradient as the eluting solvent, to afford 25 sub-fractions (D8a–D8y). The solid from fractions D8c–D8d was recrystallized from ethanol to yield the title compound (m.p. 581–583 K) as a colourless crystalline solid.

### Refinement

All hydrogen atoms were positioned geometrically (N—H = 0.88 Å and C—H = 0.98–1.0 Å) and were refined using a riding model, with *U*_iso_(H) = 1.2*U*_eq_(C). 721 Friedel pairs were used to determine the absolute configuration.

### Crystal data

**Table:**

C_32_H_49_NO_4_	*F*(000) = 1120
*M**_r_* = 511.72	*D*_x_ = 1.228 Mg m^−^^3^
Monoclinic, *C*2	Cu *K*α radiation, λ = 1.54178 Å
Hall symbol: C 2y	Cell parameters from 8407 reflections
*a* = 13.0197 (2) Å	θ = 4.2–62.5°
*b* = 6.7460 (1) Å	µ = 0.62 mm^−^^1^
*c* = 32.0674 (5) Å	*T* = 100 K
β = 100.6452 (4)°	Block, colourless
*V* = 2768.04 (7) Å^3^	0.73 × 0.15 × 0.14 mm
*Z* = 4	

### Data collection

**Table:**

Bruker SMART APEX DUO CCD area-detector diffractometer	3061 independent reflections
Radiation source: fine-focus sealed tube	3050 reflections with *I* > 2σ(*I*)
graphite	*R*_int_ = 0.018
φ and ω scans	θ_max_ = 62.5°, θ_min_ = 4.2°
Absorption correction: multi-scan (*SADABS*; Bruker, 2009)	*h* = −14→14
*T*_min_ = 0.659, *T*_max_ = 0.917	*k* = −7→5
8407 measured reflections	*l* = −36→36

### Refinement

**Table:**

Refinement on *F*^2^	Secondary atom site location: difference Fourier map
Least-squares matrix: full	Hydrogen site location: inferred from neighbouring sites
*R*[*F*^2^ > 2σ(*F*^2^)] = 0.033	H-atom parameters constrained
*wR*(*F*^2^) = 0.087	*w* = 1/[σ^2^(*F*_o_^2^) + (0.0534*P*)^2^ + 1.724*P*] where *P* = (*F*_o_^2^ + 2*F*_c_^2^)/3
*S* = 1.02	(Δ/σ)_max_ = 0.001
3061 reflections	Δρ_max_ = 0.32 e Å^−^^3^
342 parameters	Δρ_min_ = −0.37 e Å^−^^3^
1 restraint	Absolute structure: Flack (1983), 721 Friedel pairs
Primary atom site location: structure-invariant direct methods	Flack parameter: 0.1 (2)

### Special details

**Table:**

Experimental. The crystal was placed in the cold stream of an Oxford Cryosystems Cobra open-flow nitrogen cryostat (Cosier & Glazer, 1986) operating at 100.0 (1) K.
Geometry. All s.u.'s (except the s.u. in the dihedral angle between two l.s. planes) are estimated using the full covariance matrix. The cell s.u.'s are taken into account individually in the estimation of s.u.'s in distances, angles and torsion angles; correlations between s.u.'s in cell parameters are only used when they are defined by crystal symmetry. An approximate (isotropic) treatment of cell s.u.'s is used for estimating s.u.'s involving l.s. planes.
Refinement. Refinement of F^2^ against ALL reflections. The weighted R-factor wR and goodness of fit S are based on F^2^, conventional R-factors R are based on F, with F set to zero for negative F^2^. The threshold expression of F^2^ > 2σ(F^2^) is used only for calculating R-factors(gt) etc. and is not relevant to the choice of reflections for refinement. R-factors based on F^2^ are statistically about twice as large as those based on F, and R- factors based on ALL data will be even larger.

### Fractional atomic coordinates and isotropic or equivalent isotropic displacement parameters (Å^2^)

**Table:**

	*x*	*y*	*z*	*U*_iso_*/*U*_eq_	
O1	0.32106 (11)	−0.0737 (2)	0.17064 (4)	0.0246 (3)	
O2	0.62607 (11)	0.4500 (2)	0.44068 (4)	0.0270 (4)	
O3	0.67821 (13)	0.7573 (3)	0.42805 (4)	0.0378 (4)	
O4	0.19103 (13)	−0.2023 (3)	0.12291 (5)	0.0355 (4)	
N1	0.55624 (13)	0.2185 (4)	0.21012 (6)	0.0339 (5)	
H1	0.5937	0.3172	0.2030	0.041*	
C1	0.24506 (16)	−0.0616 (4)	0.13528 (6)	0.0246 (5)	
C2	0.24394 (14)	0.1455 (3)	0.11795 (6)	0.0205 (4)	
C3	0.21283 (15)	0.1641 (4)	0.06963 (6)	0.0218 (4)	
H3A	0.1363	0.1478	0.0611	0.026*	
H3B	0.2472	0.0582	0.0558	0.026*	
C4	0.24546 (15)	0.3678 (4)	0.05499 (6)	0.0243 (5)	
H4A	0.2039	0.4715	0.0661	0.029*	
H4B	0.2283	0.3734	0.0236	0.029*	
C5	0.36277 (16)	0.4152 (4)	0.06945 (6)	0.0256 (5)	
C6	0.39041 (15)	0.4014 (4)	0.11863 (6)	0.0234 (5)	
H6A	0.4663	0.4229	0.1283	0.028*	
H6B	0.3525	0.5055	0.1314	0.028*	
C7	0.36000 (15)	0.1978 (3)	0.13284 (6)	0.0196 (4)	
H7A	0.3993	0.1012	0.1181	0.023*	
C8	0.37372 (15)	0.1226 (3)	0.17907 (6)	0.0197 (4)	
C9	0.31546 (14)	0.2417 (3)	0.20917 (5)	0.0169 (4)	
C10	0.19759 (14)	0.2301 (4)	0.19046 (5)	0.0217 (4)	
H10A	0.1599	0.3287	0.2049	0.026*	
H10B	0.1713	0.0969	0.1961	0.026*	
C11	0.17391 (14)	0.2695 (4)	0.14213 (5)	0.0214 (4)	
H11A	0.1845	0.4120	0.1369	0.026*	
H11B	0.0997	0.2377	0.1309	0.026*	
C12	0.48492 (15)	0.0697 (4)	0.19851 (6)	0.0282 (5)	
H12A	0.5131	−0.0480	0.1854	0.034*	
C13	0.52441 (15)	0.0924 (4)	0.24330 (6)	0.0304 (6)	
H13A	0.5763	−0.0087	0.2568	0.036*	
C14	0.46077 (14)	0.1942 (3)	0.27216 (5)	0.0184 (4)	
H14A	0.4692	0.3391	0.2672	0.022*	
C15	0.50418 (14)	0.1640 (3)	0.32060 (6)	0.0194 (4)	
C16	0.61602 (14)	0.2523 (4)	0.32994 (6)	0.0234 (5)	
H16A	0.6639	0.1625	0.3183	0.028*	
H16B	0.6158	0.3814	0.3153	0.028*	
C17	0.65743 (15)	0.2823 (4)	0.37752 (6)	0.0243 (5)	
H17A	0.6627	0.1528	0.3923	0.029*	
H17B	0.7280	0.3423	0.3818	0.029*	
C18	0.58362 (15)	0.4169 (3)	0.39561 (5)	0.0230 (5)	
H18A	0.5801	0.5472	0.3805	0.028*	
C19	0.47224 (15)	0.3357 (3)	0.39178 (6)	0.0222 (5)	
C20	0.43358 (14)	0.2950 (3)	0.34356 (5)	0.0192 (4)	
H20A	0.4339	0.4281	0.3299	0.023*	
C21	0.31921 (14)	0.2264 (4)	0.33240 (5)	0.0218 (4)	
H21A	0.3146	0.0843	0.3396	0.026*	
H21B	0.2765	0.3026	0.3493	0.026*	
C22	0.27630 (14)	0.2569 (4)	0.28508 (5)	0.0217 (4)	
H22A	0.2741	0.4007	0.2788	0.026*	
H22B	0.2038	0.2061	0.2785	0.026*	
C23	0.34200 (14)	0.1521 (3)	0.25602 (6)	0.0183 (4)	
C24	0.66861 (15)	0.6269 (3)	0.45241 (6)	0.0240 (5)	
C25	0.7020 (2)	0.6407 (4)	0.49973 (7)	0.0368 (6)	
H25A	0.6668	0.7533	0.5104	0.055*	
H25B	0.7778	0.6598	0.5068	0.055*	
H25C	0.6831	0.5182	0.5129	0.055*	
C26	0.43081 (16)	0.2772 (4)	0.04793 (6)	0.0336 (6)	
H26A	0.5047	0.3078	0.0582	0.050*	
H26B	0.4140	0.2964	0.0171	0.050*	
H26C	0.4171	0.1391	0.0547	0.050*	
C27	0.38219 (18)	0.6281 (4)	0.05690 (6)	0.0342 (6)	
H27A	0.4548	0.6647	0.0684	0.051*	
H27B	0.3349	0.7174	0.0684	0.051*	
H27C	0.3693	0.6391	0.0259	0.051*	
C28	0.34752 (16)	0.4625 (3)	0.20954 (6)	0.0227 (5)	
H28A	0.3000	0.5337	0.1873	0.034*	
H28B	0.4192	0.4731	0.2043	0.034*	
H28C	0.3438	0.5205	0.2372	0.034*	
C29	0.51138 (16)	−0.0556 (4)	0.33404 (6)	0.0255 (5)	
H29A	0.5653	−0.0711	0.3595	0.038*	
H29B	0.5296	−0.1362	0.3110	0.038*	
H29C	0.4438	−0.0992	0.3401	0.038*	
C30	0.40528 (17)	0.5014 (4)	0.40603 (6)	0.0296 (5)	
H30A	0.4392	0.5506	0.4339	0.044*	
H30B	0.3360	0.4490	0.4078	0.044*	
H30C	0.3980	0.6101	0.3854	0.044*	
C31	0.46755 (16)	0.1551 (4)	0.42054 (6)	0.0270 (5)	
H31A	0.4845	0.1964	0.4503	0.040*	
H31B	0.5181	0.0554	0.4150	0.040*	
H31C	0.3971	0.0984	0.4146	0.040*	
C32	0.31756 (17)	−0.0706 (3)	0.25724 (6)	0.0246 (5)	
H32A	0.3163	−0.1105	0.2865	0.037*	
H32B	0.3716	−0.1460	0.2465	0.037*	
H32C	0.2493	−0.0971	0.2395	0.037*	

### Atomic displacement parameters (Å^2^)

**Table:**

	*U*^11^	*U*^22^	*U*^33^	*U*^12^	*U*^13^	*U*^23^
O1	0.0363 (8)	0.0157 (8)	0.0206 (6)	0.0029 (7)	0.0019 (5)	−0.0023 (6)
O2	0.0373 (8)	0.0246 (9)	0.0164 (6)	−0.0043 (7)	−0.0025 (5)	0.0014 (6)
O3	0.0515 (9)	0.0313 (10)	0.0270 (7)	−0.0149 (9)	−0.0024 (6)	0.0025 (8)
O4	0.0508 (9)	0.0236 (10)	0.0292 (7)	−0.0085 (8)	0.0001 (7)	−0.0039 (7)
N1	0.0171 (8)	0.0479 (14)	0.0367 (9)	−0.0027 (9)	0.0048 (7)	−0.0081 (10)
C1	0.0309 (10)	0.0235 (13)	0.0194 (9)	−0.0020 (10)	0.0045 (7)	−0.0024 (9)
C2	0.0205 (9)	0.0193 (11)	0.0212 (9)	0.0002 (9)	0.0024 (7)	−0.0009 (9)
C3	0.0224 (9)	0.0237 (12)	0.0180 (9)	−0.0003 (9)	0.0001 (7)	−0.0030 (8)
C4	0.0265 (10)	0.0295 (13)	0.0157 (8)	−0.0002 (10)	0.0008 (7)	0.0017 (9)
C5	0.0271 (10)	0.0305 (14)	0.0188 (9)	−0.0020 (10)	0.0033 (7)	0.0029 (9)
C6	0.0196 (9)	0.0314 (14)	0.0185 (9)	−0.0025 (9)	0.0017 (7)	0.0008 (9)
C7	0.0203 (9)	0.0213 (13)	0.0173 (8)	0.0031 (8)	0.0038 (7)	−0.0021 (8)
C8	0.0231 (9)	0.0150 (12)	0.0210 (9)	0.0025 (9)	0.0043 (7)	−0.0018 (8)
C9	0.0182 (9)	0.0143 (11)	0.0183 (8)	0.0028 (8)	0.0035 (7)	−0.0014 (8)
C10	0.0199 (9)	0.0254 (12)	0.0200 (9)	0.0040 (9)	0.0043 (7)	−0.0003 (9)
C11	0.0185 (8)	0.0236 (12)	0.0205 (9)	0.0019 (9)	−0.0003 (7)	−0.0001 (9)
C12	0.0246 (10)	0.0370 (15)	0.0241 (10)	0.0137 (11)	0.0074 (8)	−0.0003 (10)
C13	0.0234 (9)	0.0482 (17)	0.0193 (9)	0.0122 (11)	0.0035 (8)	−0.0008 (10)
C14	0.0198 (9)	0.0177 (12)	0.0177 (8)	0.0022 (8)	0.0036 (7)	−0.0008 (8)
C15	0.0205 (9)	0.0210 (12)	0.0163 (8)	0.0031 (9)	0.0025 (7)	−0.0002 (8)
C16	0.0205 (9)	0.0301 (13)	0.0195 (9)	0.0031 (9)	0.0034 (7)	0.0017 (9)
C17	0.0225 (9)	0.0295 (13)	0.0195 (9)	−0.0016 (10)	0.0006 (7)	0.0038 (9)
C18	0.0318 (10)	0.0221 (13)	0.0135 (8)	−0.0025 (10)	−0.0005 (7)	0.0010 (9)
C19	0.0280 (10)	0.0217 (12)	0.0176 (9)	0.0017 (9)	0.0059 (7)	−0.0002 (8)
C20	0.0224 (9)	0.0182 (12)	0.0172 (8)	0.0023 (9)	0.0041 (7)	0.0020 (8)
C21	0.0217 (9)	0.0256 (12)	0.0194 (9)	0.0003 (9)	0.0072 (7)	−0.0019 (9)
C22	0.0193 (8)	0.0243 (12)	0.0216 (9)	0.0035 (9)	0.0042 (7)	−0.0007 (9)
C23	0.0194 (9)	0.0168 (11)	0.0191 (9)	0.0013 (8)	0.0044 (7)	−0.0022 (8)
C24	0.0228 (9)	0.0257 (13)	0.0228 (10)	0.0004 (10)	0.0019 (7)	0.0003 (10)
C25	0.0522 (13)	0.0316 (15)	0.0242 (10)	−0.0048 (12)	0.0009 (9)	−0.0020 (10)
C26	0.0277 (10)	0.0529 (18)	0.0213 (9)	0.0005 (12)	0.0069 (8)	−0.0001 (11)
C27	0.0378 (11)	0.0404 (16)	0.0229 (10)	−0.0111 (12)	0.0018 (9)	0.0070 (10)
C28	0.0300 (10)	0.0180 (12)	0.0192 (9)	0.0017 (9)	0.0021 (7)	−0.0006 (9)
C29	0.0321 (10)	0.0210 (12)	0.0213 (9)	0.0071 (10)	−0.0005 (8)	−0.0009 (9)
C30	0.0349 (11)	0.0317 (15)	0.0215 (9)	0.0058 (10)	0.0038 (8)	−0.0042 (9)
C31	0.0317 (10)	0.0299 (13)	0.0195 (9)	−0.0041 (10)	0.0053 (8)	0.0015 (9)
C32	0.0342 (11)	0.0181 (12)	0.0210 (9)	−0.0048 (10)	0.0033 (8)	0.0016 (9)

### Geometric parameters (Å, °)

**Table:**

O1—C1	1.363 (2)	C15—C20	1.555 (3)
O1—C8	1.493 (3)	C16—C17	1.535 (2)
O2—C24	1.340 (3)	C16—H16A	0.9900
O2—C18	1.466 (2)	C16—H16B	0.9900
O3—C24	1.198 (3)	C17—C18	1.514 (3)
O4—C1	1.204 (3)	C17—H17A	0.9900
N1—C12	1.371 (3)	C17—H17B	0.9900
N1—C13	1.480 (3)	C18—C19	1.534 (3)
N1—H1	0.8800	C18—H18A	1.0000
C1—C2	1.502 (3)	C19—C31	1.536 (3)
C2—C3	1.533 (2)	C19—C30	1.538 (3)
C2—C7	1.539 (3)	C19—C20	1.560 (2)
C2—C11	1.547 (3)	C20—C21	1.537 (2)
C3—C4	1.537 (3)	C20—H20A	1.0000
C3—H3A	0.9900	C21—C22	1.531 (2)
C3—H3B	0.9900	C21—H21A	0.9900
C4—C5	1.546 (3)	C21—H21B	0.9900
C4—H4A	0.9900	C22—C23	1.547 (3)
C4—H4B	0.9900	C22—H22A	0.9900
C5—C27	1.525 (4)	C22—H22B	0.9900
C5—C26	1.534 (3)	C23—C32	1.538 (3)
C5—C6	1.554 (2)	C24—C25	1.502 (3)
C6—C7	1.522 (3)	C25—H25A	0.9800
C6—H6A	0.9900	C25—H25B	0.9800
C6—H6B	0.9900	C25—H25C	0.9800
C7—C8	1.546 (2)	C26—H26A	0.9800
C7—H7A	1.0000	C26—H26B	0.9800
C8—C12	1.510 (3)	C26—H26C	0.9800
C8—C9	1.557 (2)	C27—H27A	0.9800
C9—C10	1.543 (2)	C27—H27B	0.9800
C9—C28	1.546 (3)	C27—H27C	0.9800
C9—C23	1.597 (2)	C28—H28A	0.9800
C10—C11	1.546 (2)	C28—H28B	0.9800
C10—H10A	0.9900	C28—H28C	0.9800
C10—H10B	0.9900	C29—H29A	0.9800
C11—H11A	0.9900	C29—H29B	0.9800
C11—H11B	0.9900	C29—H29C	0.9800
C12—C13	1.442 (3)	C30—H30A	0.9800
C12—H12A	1.0000	C30—H30B	0.9800
C13—C14	1.516 (3)	C30—H30C	0.9800
C13—H13A	1.0000	C31—H31A	0.9800
C14—C23	1.563 (2)	C31—H31B	0.9800
C14—C15	1.565 (2)	C31—H31C	0.9800
C14—H14A	1.0000	C32—H32A	0.9800
C15—C29	1.541 (3)	C32—H32B	0.9800
C15—C16	1.550 (3)	C32—H32C	0.9800
			
C1—O1—C8	109.63 (16)	C15—C16—H16B	109.0
C24—O2—C18	118.26 (16)	H16A—C16—H16B	107.8
C12—N1—C13	60.61 (15)	C18—C17—C16	109.20 (15)
C12—N1—H1	149.7	C18—C17—H17A	109.8
C13—N1—H1	149.7	C16—C17—H17A	109.8
O4—C1—O1	121.1 (2)	C18—C17—H17B	109.8
O4—C1—C2	130.24 (18)	C16—C17—H17B	109.8
O1—C1—C2	108.64 (18)	H17A—C17—H17B	108.3
C1—C2—C3	115.65 (17)	O2—C18—C17	108.59 (15)
C1—C2—C7	99.01 (16)	O2—C18—C19	108.18 (14)
C3—C2—C7	110.84 (15)	C17—C18—C19	114.38 (18)
C1—C2—C11	106.48 (16)	O2—C18—H18A	108.5
C3—C2—C11	113.10 (16)	C17—C18—H18A	108.5
C7—C2—C11	110.87 (16)	C19—C18—H18A	108.5
C2—C3—C4	110.22 (16)	C18—C19—C31	112.16 (16)
C2—C3—H3A	109.6	C18—C19—C30	107.22 (18)
C4—C3—H3A	109.6	C31—C19—C30	108.08 (16)
C2—C3—H3B	109.6	C18—C19—C20	105.63 (14)
C4—C3—H3B	109.6	C31—C19—C20	114.48 (18)
H3A—C3—H3B	108.1	C30—C19—C20	109.00 (16)
C3—C4—C5	114.01 (17)	C21—C20—C15	110.54 (16)
C3—C4—H4A	108.8	C21—C20—C19	113.90 (14)
C5—C4—H4A	108.8	C15—C20—C19	117.30 (15)
C3—C4—H4B	108.8	C21—C20—H20A	104.5
C5—C4—H4B	108.8	C15—C20—H20A	104.5
H4A—C4—H4B	107.6	C19—C20—H20A	104.5
C27—C5—C26	108.38 (18)	C22—C21—C20	110.53 (15)
C27—C5—C4	108.57 (18)	C22—C21—H21A	109.5
C26—C5—C4	111.10 (18)	C20—C21—H21A	109.5
C27—C5—C6	107.80 (18)	C22—C21—H21B	109.5
C26—C5—C6	112.04 (17)	C20—C21—H21B	109.5
C4—C5—C6	108.83 (15)	H21A—C21—H21B	108.1
C7—C6—C5	109.49 (17)	C21—C22—C23	113.23 (16)
C7—C6—H6A	109.8	C21—C22—H22A	108.9
C5—C6—H6A	109.8	C23—C22—H22A	108.9
C7—C6—H6B	109.8	C21—C22—H22B	108.9
C5—C6—H6B	109.8	C23—C22—H22B	108.9
H6A—C6—H6B	108.2	H22A—C22—H22B	107.7
C6—C7—C2	114.13 (17)	C32—C23—C22	106.92 (17)
C6—C7—C8	126.37 (17)	C32—C23—C14	111.44 (17)
C2—C7—C8	98.92 (15)	C22—C23—C14	109.92 (15)
C6—C7—H7A	105.2	C32—C23—C9	112.57 (16)
C2—C7—H7A	105.2	C22—C23—C9	110.26 (16)
C8—C7—H7A	105.2	C14—C23—C9	105.77 (14)
O1—C8—C12	103.81 (17)	O3—C24—O2	123.95 (18)
O1—C8—C7	98.61 (14)	O3—C24—C25	124.6 (2)
C12—C8—C7	113.93 (15)	O2—C24—C25	111.48 (19)
O1—C8—C9	108.06 (14)	C24—C25—H25A	109.5
C12—C8—C9	113.95 (15)	C24—C25—H25B	109.5
C7—C8—C9	116.24 (17)	H25A—C25—H25B	109.5
C10—C9—C28	107.43 (17)	C24—C25—H25C	109.5
C10—C9—C8	107.23 (15)	H25A—C25—H25C	109.5
C28—C9—C8	109.88 (15)	H25B—C25—H25C	109.5
C10—C9—C23	111.93 (15)	C5—C26—H26A	109.5
C28—C9—C23	110.24 (16)	C5—C26—H26B	109.5
C8—C9—C23	110.04 (16)	H26A—C26—H26B	109.5
C9—C10—C11	112.39 (14)	C5—C26—H26C	109.5
C9—C10—H10A	109.1	H26A—C26—H26C	109.5
C11—C10—H10A	109.1	H26B—C26—H26C	109.5
C9—C10—H10B	109.1	C5—C27—H27A	109.5
C11—C10—H10B	109.1	C5—C27—H27B	109.5
H10A—C10—H10B	107.9	H27A—C27—H27B	109.5
C10—C11—C2	112.49 (16)	C5—C27—H27C	109.5
C10—C11—H11A	109.1	H27A—C27—H27C	109.5
C2—C11—H11A	109.1	H27B—C27—H27C	109.5
C10—C11—H11B	109.1	C9—C28—H28A	109.5
C2—C11—H11B	109.1	C9—C28—H28B	109.5
H11A—C11—H11B	107.8	H28A—C28—H28B	109.5
N1—C12—C13	63.43 (16)	C9—C28—H28C	109.5
N1—C12—C8	119.3 (2)	H28A—C28—H28C	109.5
C13—C12—C8	121.65 (17)	H28B—C28—H28C	109.5
N1—C12—H12A	114.4	C15—C29—H29A	109.5
C13—C12—H12A	114.4	C15—C29—H29B	109.5
C8—C12—H12A	114.4	H29A—C29—H29B	109.5
C12—C13—N1	55.96 (15)	C15—C29—H29C	109.5
C12—C13—C14	121.46 (17)	H29A—C29—H29C	109.5
N1—C13—C14	115.9 (2)	H29B—C29—H29C	109.5
C12—C13—H13A	116.5	C19—C30—H30A	109.5
N1—C13—H13A	116.5	C19—C30—H30B	109.5
C14—C13—H13A	116.5	H30A—C30—H30B	109.5
C13—C14—C23	109.54 (15)	C19—C30—H30C	109.5
C13—C14—C15	114.15 (16)	H30A—C30—H30C	109.5
C23—C14—C15	117.30 (15)	H30B—C30—H30C	109.5
C13—C14—H14A	104.8	C19—C31—H31A	109.5
C23—C14—H14A	104.8	C19—C31—H31B	109.5
C15—C14—H14A	104.8	H31A—C31—H31B	109.5
C29—C15—C16	107.99 (18)	C19—C31—H31C	109.5
C29—C15—C20	115.13 (16)	H31A—C31—H31C	109.5
C16—C15—C20	107.86 (17)	H31B—C31—H31C	109.5
C29—C15—C14	113.26 (16)	C23—C32—H32A	109.5
C16—C15—C14	107.20 (15)	C23—C32—H32B	109.5
C20—C15—C14	105.03 (15)	H32A—C32—H32B	109.5
C17—C16—C15	112.80 (15)	C23—C32—H32C	109.5
C17—C16—H16A	109.0	H32A—C32—H32C	109.5
C15—C16—H16A	109.0	H32B—C32—H32C	109.5
C17—C16—H16B	109.0		
			
C8—O1—C1—O4	−178.70 (18)	C12—N1—C13—C14	−111.9 (2)
C8—O1—C1—C2	0.5 (2)	C12—C13—C14—C23	33.2 (3)
O4—C1—C2—C3	−33.0 (3)	N1—C13—C14—C23	97.6 (2)
O1—C1—C2—C3	147.89 (16)	C12—C13—C14—C15	167.0 (2)
O4—C1—C2—C7	−151.4 (2)	N1—C13—C14—C15	−128.61 (18)
O1—C1—C2—C7	29.49 (19)	C13—C14—C15—C29	−58.5 (2)
O4—C1—C2—C11	93.6 (2)	C23—C14—C15—C29	71.6 (2)
O1—C1—C2—C11	−85.54 (19)	C13—C14—C15—C16	60.5 (2)
C1—C2—C3—C4	−163.28 (17)	C23—C14—C15—C16	−169.39 (18)
C7—C2—C3—C4	−51.7 (2)	C13—C14—C15—C20	175.07 (19)
C11—C2—C3—C4	73.6 (2)	C23—C14—C15—C20	−54.8 (2)
C2—C3—C4—C5	55.2 (2)	C29—C15—C16—C17	−72.5 (2)
C3—C4—C5—C27	−174.19 (16)	C20—C15—C16—C17	52.5 (2)
C3—C4—C5—C26	66.7 (2)	C14—C15—C16—C17	165.10 (18)
C3—C4—C5—C6	−57.1 (2)	C15—C16—C17—C18	−57.9 (3)
C27—C5—C6—C7	173.35 (17)	C24—O2—C18—C17	106.1 (2)
C26—C5—C6—C7	−67.5 (2)	C24—O2—C18—C19	−129.18 (19)
C4—C5—C6—C7	55.8 (2)	C16—C17—C18—O2	−177.98 (17)
C5—C6—C7—C2	−57.1 (2)	C16—C17—C18—C19	61.1 (2)
C5—C6—C7—C8	−179.84 (18)	O2—C18—C19—C31	−52.2 (2)
C1—C2—C7—C6	177.12 (15)	C17—C18—C19—C31	69.0 (2)
C3—C2—C7—C6	55.2 (2)	O2—C18—C19—C30	66.3 (2)
C11—C2—C7—C6	−71.3 (2)	C17—C18—C19—C30	−172.55 (16)
C1—C2—C7—C8	−46.17 (17)	O2—C18—C19—C20	−177.56 (17)
C3—C2—C7—C8	−168.12 (18)	C17—C18—C19—C20	−56.4 (2)
C11—C2—C7—C8	65.4 (2)	C29—C15—C20—C21	−64.4 (2)
C1—O1—C8—C12	−147.48 (15)	C16—C15—C20—C21	175.02 (16)
C1—O1—C8—C7	−30.11 (18)	C14—C15—C20—C21	60.9 (2)
C1—O1—C8—C9	91.20 (17)	C29—C15—C20—C19	68.5 (2)
C6—C7—C8—O1	175.33 (17)	C16—C15—C20—C19	−52.1 (2)
C2—C7—C8—O1	46.32 (17)	C14—C15—C20—C19	−166.21 (17)
C6—C7—C8—C12	−75.3 (3)	C18—C19—C20—C21	−175.71 (18)
C2—C7—C8—C12	155.67 (19)	C31—C19—C20—C21	60.4 (2)
C6—C7—C8—C9	60.2 (2)	C30—C19—C20—C21	−60.8 (2)
C2—C7—C8—C9	−68.8 (2)	C18—C19—C20—C15	53.0 (2)
O1—C8—C9—C10	−48.83 (19)	C31—C19—C20—C15	−71.0 (2)
C12—C8—C9—C10	−163.63 (19)	C30—C19—C20—C15	167.88 (18)
C7—C8—C9—C10	60.8 (2)	C15—C20—C21—C22	−64.6 (2)
O1—C8—C9—C28	−165.30 (14)	C19—C20—C21—C22	160.85 (18)
C12—C8—C9—C28	79.9 (2)	C20—C21—C22—C23	56.1 (2)
C7—C8—C9—C28	−55.6 (2)	C21—C22—C23—C32	74.7 (2)
O1—C8—C9—C23	73.13 (18)	C21—C22—C23—C14	−46.4 (2)
C12—C8—C9—C23	−41.7 (2)	C21—C22—C23—C9	−162.63 (17)
C7—C8—C9—C23	−177.21 (16)	C13—C14—C23—C32	62.1 (2)
C28—C9—C10—C11	72.3 (2)	C15—C14—C23—C32	−70.1 (2)
C8—C9—C10—C11	−45.8 (2)	C13—C14—C23—C22	−179.53 (19)
C23—C9—C10—C11	−166.56 (18)	C15—C14—C23—C22	48.3 (2)
C9—C10—C11—C2	49.1 (3)	C13—C14—C23—C9	−60.5 (2)
C1—C2—C11—C10	45.1 (2)	C15—C14—C23—C9	167.27 (17)
C3—C2—C11—C10	173.17 (18)	C10—C9—C23—C32	63.8 (2)
C7—C2—C11—C10	−61.6 (2)	C28—C9—C23—C32	−176.72 (16)
C13—N1—C12—C8	113.1 (2)	C8—C9—C23—C32	−55.4 (2)
O1—C8—C12—N1	179.68 (16)	C10—C9—C23—C22	−55.5 (2)
C7—C8—C12—N1	73.5 (2)	C28—C9—C23—C22	64.0 (2)
C9—C8—C12—N1	−63.0 (2)	C8—C9—C23—C22	−174.65 (16)
O1—C8—C12—C13	−105.2 (2)	C10—C9—C23—C14	−174.31 (17)
C7—C8—C12—C13	148.7 (2)	C28—C9—C23—C14	−54.80 (19)
C9—C8—C12—C13	12.1 (3)	C8—C9—C23—C14	66.6 (2)
C8—C12—C13—N1	−109.5 (3)	C18—O2—C24—O3	−3.3 (3)
N1—C12—C13—C14	101.7 (3)	C18—O2—C24—C25	176.38 (17)
C8—C12—C13—C14	−7.8 (4)		
